# ADHD: Braun et al. Respond

**DOI:** 10.1289/ehp.10274R

**Published:** 2007-08

**Authors:** Joe M. Braun, Bruce P. Lanphear, Robert S. Kahn, Tanya Froehlich, Peggy Auinger

**Affiliations:** Department of Epidemiology, University of North Carolina-Chapel Hill, Chapel Hill, North Carolina, E-mail: jmbraun@unc.edu; Department of Pediatrics, Cincinnati Children’s Hospital Medical Center, Cincinnati, Ohio, E-mail: bruce.lanphear@chmcc.org; Department of Pediatrics, University of Rochester School of Medicine, Rochester, New York

We appreciate the comments of Brondum, and Konofal and Cortese, and the opportunity to clarify our results (Braun et al. 2006). It is common practice to select variables with a *p*-value of 0.2 for inclusion in multivariable models (Katz 1999). Although the association of blood lead levels and ADHD appeared “tenuous” in bivariate analysis (i.e., *p* = 0.19), this was largely an artifact of our decision to categorize blood lead levels. When we entered lead into our multivariable analysis as a continuous variable, we found a 1.2-fold increased odds [95% confidence interval (CI), 1.0–1.4; *p* = 0.02] of ADHD for each 1.0-μg/dL increase in blood lead levels. The blood lead quintiles were not divided into exactly equal sample sizes because we used weighted percentages to categorize the data. We decided *a priori* to present the analysis in quintiles to make the results easier to interpret and also to illustrate any dose–response relationships for blood lead levels and ADHD.

As we noted in the “Discussion” of our article (Braun et al. 2006), a limitation of our study was the inability to adjust for parental psychopathology. This is an unfortunate trade-off when using a large nationally representative survey. In other studies, prenatal tobacco exposure has been shown to be a risk factor for the development of ADHD after controlling for parental psychopathology (Mick et al. 2002; Weissman et al. 1999). Although there is considerable experimental and epidemiologic evidence linking lead exposure with behaviors consistent with ADHD, future studies of childhood lead exposure will need to confirm our results by accounting for parental psychopathology and other potential confounders.

The hypothesis proposed by Konofal and Cortese—that iron deficiency may play a role in symptom severity among children with ADHD—is intriguing. Indeed, it was their original research that prompted us to incorporate ferritin as a measure of iron status (Konofal et al. 2004). It is certainly plausible that iron deficiency may confound or modify the effects of environmental lead exposure on ADHD in children. Alternatively, lead exposure may act as a confounder or modifier for the observed effects of iron deficiency with ADHD. Unfortunately, we were not able to examine whether ferritin (or other indicators of iron status) was associated with ADHD symptom severity using the National Health and Nutrition Examination Survey. Nor did we specifically test for an association between iron deficiency and ADHD. Although iron or other micronutrient supplementation may protect children from lead toxicity, recent evidence from a double-blind randomized trial (Kordas et al. 2005) suggests that iron and zinc supplementation did not appreciably lower blood lead levels or improve child behavior, as measured by the Conners Rating Scales. However, Kordas et al. included only children without anemia in their trial.
